# Ecological niche models for the assessment of site suitability of sea cucumbers and sea urchins in China

**DOI:** 10.1038/s41598-022-17004-6

**Published:** 2022-07-27

**Authors:** Jiangnan Sun, Yushi Yu, Zihe Zhao, Donghong Yin, Yaqing Chang, Chong Zhao

**Affiliations:** Key Laboratory of Mariculture and Stock Enhancement in North China’s Sea, Ministry of Agriculture and Rural Affairs, Dalian Ocean University, Dalian, 116023 China

**Keywords:** Agroecology, Ecology

## Abstract

In the present study, the maximum entropy model (MaxEnt) based on the data of sea surface temperature (SST) and published information was used to assess the site suitability for the aquaculture expansion of the sea cucumber *Apostichopus japonicus* and the sea urchin *Strongylocentrotus intermedius* in China. According to the current assessment, the coastal areas of Hebei province and Tianjin have great prospects for *A. japonicus* aquaculture, while is currently being underutilized. In the south, more than 94% of the coastal areas in Zhejiang, Fujian, and Guangdong provinces are suitable for the growth of *A. japonicus* for six months, especially the coastal areas of Lianjiang, Changle, Fuqing and Putian in Fujian province. The water temperatures in more than 94% of China's coastal areas are higher than 25 °C in July and August, which probably results in the mortality of *S. intermedius* in aquaculture. This clearly indicates that high water temperature is the bottleneck of *S. intermedius* aquaculture and well explains the limited expansion of this commercially important exotic species since the introduction in 1989. We suggest a new aquaculture model of *S. intermedius* that extends the seed production to November to avoid the mass mortality in summer. In the south, 64% of coastal areas in Zhejiang and Fujian provinces are suitable for the transplantation of *S. intermedius* to the south. The present study suggests the ecological niche model MaxEnt based on the data of SST and published information as a new tool for the assessment of the site suitability of sea cucumbers and sea urchins in China. This provides new insights into the aquaculture expansion of native and exotic species.

## Introduction

China has the largest aquaculture sector in the world, contributing to about 60 percent of the production for both domestic and international commercial demands^1^. The expansion of aquaculture in China is thus important for the increasing global food and nutrition security^2^. China has made remarkable progress in the expansion of aquaculture. Due to the transplantation of native species and the introduction of exotic species^3^, the mariculture area in China, for example, makes a nearly 80-fold increase from 25,000 hectares in 1954 to 1.996 million hectares in 2020^4,5^. Based on the technological innovation, the aquaculture of native species not only occurs in the original sites, but also outside the range of the natural distribution^6^. Further, China is the largest producer of non-native aquatic species in the world^1^. By 2000, more than 60 exotic species had been introduced into China, contributing to 25% of the country's total production^7^. Despite the rapid development, there are still problems with aquaculture expansion in China, especially the aquaculture site selection^8^. Site selection is an important and pre-requisite step for aquaculture expansion as it provides the foundations for production, economic benefits, and the sustainability of aquaculture activities^9^. At present, the site selection largely depends on the method of trial and error for the aquaculture expansion of both native and exotic species in China, which is heavily influenced by the water environment of different regions^10^. It brings the difficulty in coordinating aquaculture expansion for fisher administrative authorities, especially considering that aquaculture in China is mainly dominated by small and medium-scale aqua-farms^7,8^. Therefore, it is important to establish an effective method to assess the site suitability in large-scale for aquaculture expansion according to water environment of different regions.

Water temperature is the most important factor for the spatial and temporal distribution of aquaculture species^11,12^. Monitoring water temperature changes in the targeted sea areas is an important method for the assessment of the survival of aquaculture species at the site^13,14^, but is not an effective approach for the large-scale management of the aquaculture expansion in China. The development of remote sensed technology ensures the wide coverage, high accuracy, and high accessibility to the data of sea surface temperature (SST)^15^. As an important indicator of sea water temperature, the data of SST has been well used to predict the resource distribution of various marine organisms, including *Thunnus albacares*^16^, *Dosidicus gigas*^17^, and *Gadus morhua*^18^. Since aquaculture is primarily conducted in coastal areas, it is directly affected by the local sea surface temperature. The SST data has been applied for the aquaculture site selection in a number of aquaculture species, including shellfish *Mizuhopecten yessoensis*^19^, *Mytilus galloprovincialis*^20^, and kelp *Saccharina japonica*^21^. Furthermore, the data of SST was successfully used to evaluate coral reef restoration, which clearly suggests the potential application of SST data in the study of marine benthos^22^. Here, we suggest an effective approach for assessing the site suitability for the aquaculture expansion in large-scale in China by analyzing remote sensed data of sea surface temperature and reviewing the published information of the targeted species.

Ecological niche models (ENMs) are a category of methods for a correlative model of the environmental conditions that meet ecological requirements of a species by using the data of species distribution and the environments^23,24^. The results predicted by ecological niche models have higher resolution, compared with the methods based on physiological limiting factors^25^. Ecological niche models based on the data of sea surface temperature have been used in fisheries. For example, the generalized additive model (GAM) and the generalized linear model (GLM) were used to predict the niche demands of *Scomber japonicus*^26^ and the maximum entropy model (MaxEnt) to predict the potential fishing areas for *Illex argentinus* and *Cololabis saira*^27,28^. In particular, MaxEnt model outperforms other models in handling with small sample sizes and is thus suitable for aquatic organisms with limitedly recorded distribution data in field^29^. Therefore, we propose that the MaxEnt is appropriate for the assessment of site suitability for the aquaculture expansion of native and exotic species in China, based on the data of SST and the species distribution data.

The sea cucumber *Apostichopus japonicus* mainly distributes in the western Pacific, including the Yellow Sea, the Sea of Japan, and the Sea of Okhotsk, with the northern boundary in Sakhalin Island area and the southern in Tanegashima of Japan^30^. In China, this commercially important species naturally distributes in the coastal regions of Liaoning, Hebei and Shandong provinces, with the southern boundary in Lianyungang, Jiangsu Province^30,31^. Water temperature varies from -2 to 30 °C in the natural distribution of *A. japonicus*^31^. Based on the outbreaks in breeding and aquaculture technology in 1998, the aquaculture of *A. japonicus* has expanded rapidly in the north of China^32^. From 2003 to 2012, the area of *A. japonicus* aquaculture in the north increased by an average of 31.0% per year^33,34^. However, the expansion speed of *A. japonicus* aquaculture in the north declined sharply to only 3.9% of the annual average expansion rate in the most recent nine years^5,34^. The transplantation to the south is a successful step in the aquaculture expansion of *A. japonicus* in China^35^. Since 2006, the aquaculture of *A. japonicus* in the south has developed rapidly. In 2020, the production of *A. japonicus* in the south has accounted for about 14% of the total production of the country^34,35^. However, the aquacultural area of *A. japonicus* in the south accounts for less than 0.8% of that of the country and the sites are very concentrated^5,36,37^. Therefore, the key to further development of *A. japonicus* aquaculture in the south is to find efficient tools to evaluate more sites suitable for the aquaculture expansion. The sea urchin *Strongylocentrotus intermedius* naturally distributes in northern regions in the Pacific coastal waters of Choshi, Chiba, the Sea of Japan around Toyama, the Korean peninsula, Sakhalin, and Vladivostok^38^. It was introduced to the coastal waters of Dalian in China for aquaculture since 1989^39^. Due to the lack of effective methods to assess the niche needs of exotic species, however, the aquaculture of *S. intermedius* is still limited to small-scale coastal areas in China in over 30 years^31,39^.

The present study used the ecological niche model MaxEnt based on the SST data to explore potential suitable sites for the aquaculture expansion of *A. japonicus* and *S. intermedius* in China. The main purposes of the present study are to investigate the feasibility of applying ecological niche models in assessment of site suitability for aquaculture expansion and discuss possible ways to expand the aquacultural scale of *A. japonicus* and *S. intermedius* in China.

## Methods

### Distribution of *A. japonicus* and *S. intermedius*

The distribution data of *A. japonicus* and *S. intermedius* was derived from the Global Biodiversity Information Facility (GBIF, http://www.gbif.org) and the literature records^40^. All data was carefully checked and deduplicated. The literature records without geographical coordinates were obtained using Google Earth (www.googleearth.com). A total of 22 samples of *A. japonicus* (8 samples from the GBIF and 14 samples from the literature records^40^) and 32 samples of *S. intermedius* (all from the GBIF) were used in the ecological niche modeling (Tables 1, 2).

**Table. 1:** Distribution data of *Apostichopus japonicus*. The distribution data of *A. japonicus* in Japan was obtained from the Global Biodiversity Information Facility (GBIF, http://www.gbif.org). The distribution data in China was obtained from literature^40^.

Country	Location	Decimal latitude	Decimal longitude
Japan	Akita	39.870000	139.810000
Japan	Akita	39.940000	139.720000
Japan	Honshu Island	34.266667	136.850000
Japan	Kanagawa	35.000000	139.000000
Japan	Kanagawa	35.141123	139.161404
Japan	Kyushu Island	31.411111	130.192222
Japan	Naoasaki	33.531797	129.679906
Japan	Tokyo Bay	35.347196	139.781252
China	Beidaihe	39.849239	119.548181
China	Changdao	37.837209	120.768637
China	Dalian	38.865251	121.557398
China	Haiyangdao	39.063592	123.136940
China	Jiaonan	35.929793	120.240497
China	Jimingdao	37.453114	122.486838
China	Lianyungang	34.777225	119.395653
China	Lidao	37.227993	122.601828
China	Longkou	37.709209	120.355467
China	Lvshun	38.786683	121.259265
China	Pingshandao	35.008112	119.895586
China	Rizhao	35.401317	119.572670
China	Rongcheng	37.149907	122.495721
China	Sangdao	37.777860	120.460846

**Table. 2:** Distribution data of *Strongylocentrotus intermedius*. The data was derived from the Global Biodiversity Information Facility (http://www.gbif.org).

Country	Location	Decimal latitude	Decimal longitude
Russia	Kamchatka	53.032574	158.627045
Russia	Pacific Ocean	46.270000	138.280000
Russia	Primor'ye	42.951544	131.874120
Russia	Primor'ye	43.031341	131.893620
Russia	Primor'ye	43.021210	131.926885
Russia	Primor'ye	42.791375	132.811740
Russia	Primor'ye	44.345581	135.836700
Russia	Primor'ye	44.953698	136.556621
Korea	Chungcheongnam-do	36.228073	126.073823
Korea	Chungcheongnam-do	36.851054	126.197033
Japan	Akita	39.920000	139.720000
Japan	Akita	39.740000	140.130000
Japan	Hakodote	41.777654	140.658779
Japan	Hokkaido	41.771628	140.667977
Japan	Hokkaido	42.413605	141.594635
Japan	Hokkaido	43.903900	144.661000
Japan	Hokkaido	43.018299	144.837010
Japan	Iwate	39.383400	141.933000
Japan	Iwate Prefecture	40.307570	142.012183
Japan	Okinawa	26.237638	127.647240
Japan	Okinawa	41.134700	140.827000
–	Nagasaki	32.742500	129.864717
–	Peter the Great Bay	42.892662	132.052293
–	Sea of Japan	46.273900	138.279000
–	Sea of Japan	43.599730	140.247664
–	Sea of Japan	43.619782	140.900768
–	Sea of Japan	43.839914	140.955960
–	Sea of Japan	44.032690	141.066343
–	Sea of Japan	44.032690	141.075542
–	Sea of Japan	42.062923	141.204323
–	Sea of Japan	44.862960	141.268714
–	Sea of Japan	44.218221	144.552631

### Sea surface temperature data processing

The data of sea surface temperature (SST) was derived from a daily Advanced Very High Resolution Radiometer (AVHRR) infrared satellite with a high resolution of 1 km (https://neo.sci.gsfc.nasa.gov). To reduce the effect of cloud cover, SST data was used to generate the monthly composite imagery. We calculated the mean, difference and standard deviation of monthly SST data from 2003 to 2020.

According to the water temperature ranges for the survival of *A. japonicus* (− 2 to 30 °C) and *S. intermedius* (− 2 to 25 °C), the coastal areas of China were divided into survivable areas and non-survivable areas^31^. Data was collected within 20 km from the coast and calculated using the geographic information processing tools ArcMap (version 10.5). The suitable water temperature range for the growth of *A. japonicus* and *S. intermedius* is from 10 to 20°C^31^. By comparing the SST data of species distributions in different months, the highest probability of being suitable for *A. japonicus* reached 91.3% in May (mean water temperature 16.0 °C), and 87.1% for *S. intermedius* in June (mean water temperature 14.7 °C). Therefore, the data of SST in May and June were selected as the reference environment in the model for *A. japonicus* and *S. intermedius*, respectively.

### Ecological niche models for *A. japonicus* and *S. intermedius* in China

The ecological niche model MaxEnt was built using MaxEnt 3.4.1. A total of 25% of the distribution data were randomly selected as the test set, and the rest of the data (75%) as the training set. We ran the algorithm either for 10 iterations or until convergence. The receiver operating characteristic curve (ROC) was utilized to evaluate the prediction accuracy of the model. AUC value > 0.9 indicates that the model predicts the true presences perfectly^41^. To clearly divide the suitable distribution areas, the coastline was classified into three levels (lowly, moderately, and highly suitable) using a Jenks’s natural breaks approach according to suitability values^42^. The results were collected and computed using ArcMap 10.5.

## Results and discussion

### Aquaculture expansion of *A. japonicus* in China

**Figure 1:**
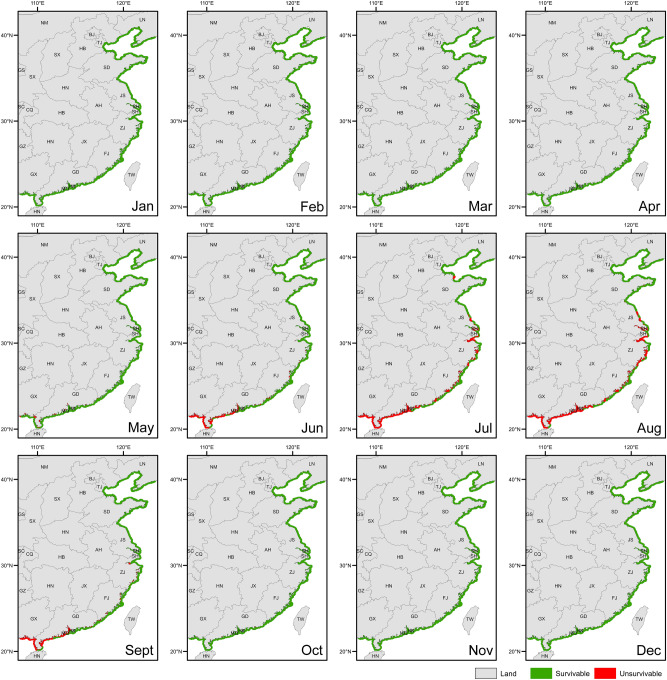
Maps of survivable areas and non-survivable areas for *Apostichopus japonicus* in China. The gray part is the land. The coastal areas marked in green indicate that sea surface temperatures are in the range where *A. japonicus* can survive. The red indicates the sea surface temperatures that exceed the tolerance of *A. japonicus*.

**Figure 2:**
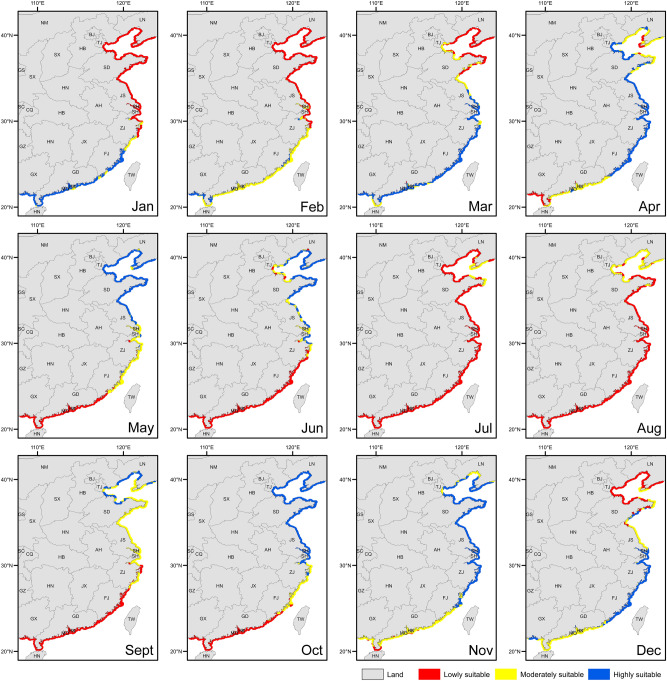
Site suitability for *Apostichopus japonicus* aquaculture in China. The blue represents the areas that are highly suitable for *A. japonicus* aquaculture. The yellow indicates that the suitability index of sea areas is medium. The red indicates that the suitability index is low.

Based on the water temperature range for the survival of *A. japonicus* (− 2 to 30 °C)^31^, we analyzed the sites where *A. japonicus* can survive along the Chinese coast in each month of the whole year (Fig. 1). The present study indicates that *A. japonicus* can survive year-round in more than 98% of the northern coastal areas (Fig. 1). Therefore, the northern coast of China is suitable for the expansion of *A. japonicus* aquaculture. At present, the area of *A. japonicus* aquaculture in the north is 2409.5 km^2^, accounting for only 4.6% of the areas that are suitable for aquaculture expansion^5^. Notably, Shandong and Liaoning provinces account for 96.1% of *A. japonicus* aquaculture areas^5^. According to the assessment results of the ecological niche model, 79% of the coastal areas of Shandong and Liaoning provinces are consistently suitable (moderate or high suitability) for the growth of *A. japonicus* for six months (April to June, September to November; Fig. 2). Furthermore, about 1128.9 km^2^ of the coastal areas (within 20 km from the coast) are suitable for *A. japonicus* to grow for a maximum of nine months (from April to December), mainly located in the northern part of the Yellow Sea (Fig. 2). Therefore, it is promising to continue the expansion of the aquaculture scale of *A. japonicus* in Shandong and Liaoning provinces. In recent years, the growth of *A. japonicus* production in Shandong has stagnated and the production in Liaoning has decreased, which leads to the decrease in the total production of *A. japonicus* in China^5^. Therefore, aquaculture of *A. japonicus* in other sites needs to be promoted. Hebei province and Tianjin are two important sites for *A. japonicus* aquaculture in the north^35,43^. Coastal water temperatures are within the temperature range for the survival of *A. japonicus* all year round in both sites (Fig. 1). More than 40% of the coastal areas in Hebei province and Tianjin are suitable for the growth of *A. japonicus* starting from March, which is earlier than that in 66.1% of Shandong province and 100% of Liaoning province (Fig. 2). Furthermore, 86.5% of the coastal areas of Hebei province and Tianjin are suitable for the growth of *A. japonicus* for six months (April to June, September to November), which is greater than that of the coastal areas of Shandong and Liaoning provinces (Fig. 2). In 2020, the aquaculture area of *A. japonicus* in Hebei province and Tianjin, most of which is in pond culture, accounts for less than 4% of the total area in northern China and only 1.7% of the local coastal area^5,43^. The present results indicate that there is still a large potential for the aquaculture expansion of *A. japonicus* in the north, especially in the coastal areas of Hebei province and Tianjin, although other factors should be further considered.

In addition to the expansion in the natural distribution areas, the transplantation of *A. japonicus* to the south is an important method for the aquaculture expansion of *A. japonicus* in China^44,45^. By the transplantation of small *A. japonicus* (~ 38 g per individual) to the coastal areas of Fujian in November, the body weight of *A. japonicus* increases by about three-fold (~ 135 g per individual) after four months of aquaculture^46^. According to the present study, more than 94% of the coastal areas of Zhejiang, Fujian, and Guangdong provinces are suitable (moderate or high suitability) for the growth of *A. japonicus* for six months (from November to the next April, Fig. 2). At present, the *A. japonicus* aquaculture in Fujian is mainly in Xiapu area, and the production accounts for 96.2% of the total production of the province^5^. According to the present assessment of the ecological niche model, the coastal areas of Lianjiang, Changle, Fuqing, and Putian in Fujian province (a total of 1424.1 km^2^) are highly suitable for *A. japonicus* aquaculture (from November to the next April, Fig. 2). It is thus feasible to largely expand the transplantation scale of *A. japonicus* to Fujian province. In addition, the southern part of Zhejiang province is another suitable site for the transplantation of *A. japonicus*. The coastal areas in the south of Taizhou in Zhejiang province are suitable for *A. japonicus* aquaculture from October until next April (Fig. 2). Fujian and Zhejiang provinces accounted for less than 1% of the *A. japonicus* aquaculture area in China, but contributed more than 14.3% to the total production in 2020^5^. This efficient aquaculture mode is thus an important step to improve the scale of *A. japonicus* aquaculture in China. The present study revealed a number of sites that are suitable for expansion of *A. japonicus* aquaculture in the south and supports the large expansion of *A. japonicus* aquaculture to the south. However, it should be noted that the present study made a preliminary assessment of potential suitable sites based on sea surface temperature. More local factors need to be considered before aquaculture expansion activities can be carried out.

### Aquaculture expansion of *S. intermedius* in China

**Figure 3:**
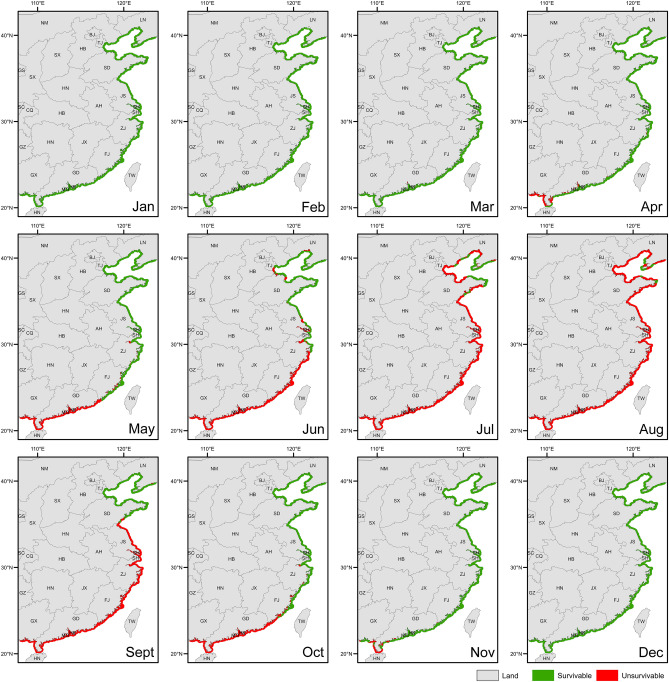
Maps of survivable areas and non-survivable areas for *Strongylocentrotus intermedius* in China. The coastal areas marked in green indicate that sea surface temperatures are in the range where *S. intermedius* can survive. The red indicates sea surface temperatures that exceed the tolerance of *S. intermedius*.

The water temperature range for the survival of *S. intermedius* is from − 2 to 25°C^31^. Temperature at 22 °C significantly affected the food consumption and gonad production of *S. intermedius*, while water temperatures above 25 °C caused mass mortality of *S. intermedius*^47–49^. According to the present study, the water temperatures in more than 94% of China's coastal areas are higher than 25 °C in July and August (Fig. 3). High water temperature in July and August is thus the bottleneck for *S. intermedius* aquaculture in China, which well explains why the aquaculture of *S. intermedius* is still limited to small-scale coastal areas in China. It is essential to break the high-water temperature bottleneck for the expansion of *S. intermedius* aquaculture, because more than 90% of the northern coastal areas are available for aquaculture after July and August (Fig. 3). Seed production of *S. intermedius* currently carries out in every October in northern China and provides individuals of 1–2 cm in test diameter for the longline culture and stock enhancement in the coming spring^50^. The subsequent longline culture and stock enhancement, however, suffer from the high water temperature, which causes mass mortality^31^. According to the present study, we suggest a new aquaculture model for *S. intermedius* that extends the seed production to every November for the longline culture and stock enhancement, which can avoid the mass mortality in summer. Notably, there are about 7537.3 km^2^ area where water temperature is lower than 25 °C all over the year, including the areas in Yantai and Weihai in Shandong province, and those in Dalian in Liaoning province (Fig. 3). These areas should be well managed for the expansion of *S. intermedius* aquaculture. In addition, heat exchange is another valuable method for the aquaculture of *S. intermedius* at high water temperatures in the areas with cold water underground.

**Figure 4:**
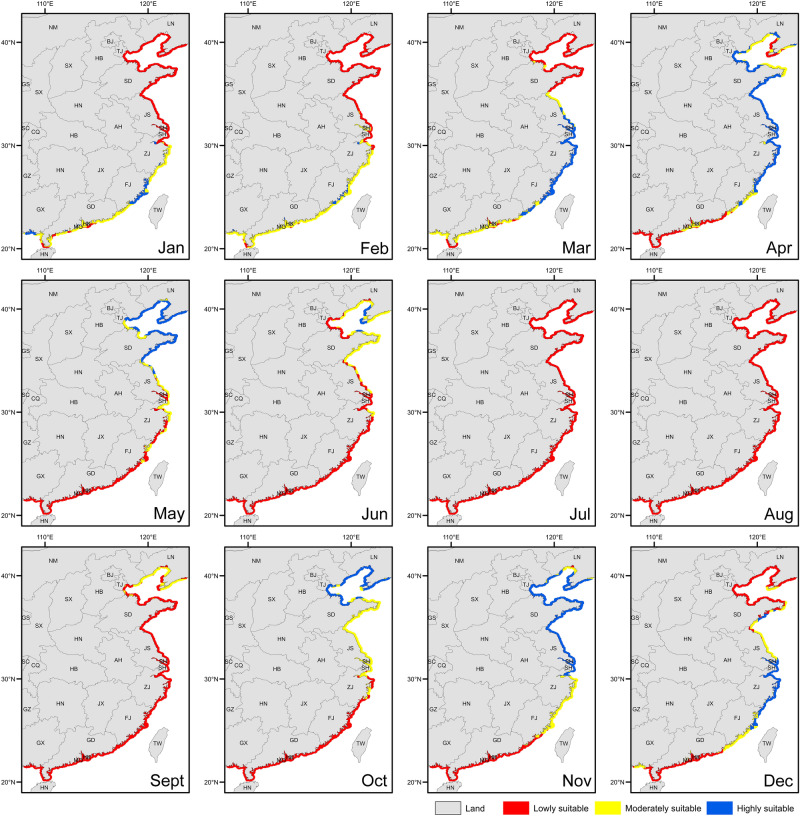
Site suitability for *Strongylocentrotus intermedius* aquaculture in China. The blue represents the areas that are highly suitable for *S. intermedius* aquaculture. The yellow indicates that the suitability index is medium. The red indicates that the suitability index is low.

Provided that the bottleneck is broken, not only *S. intermedius* aquaculture can be greatly expanded in the north, but also *S. intermedius* is suitable for the transplantation to the south. Sixty-four percent of the coastal areas in Zhejiang and Fujian provinces are suitable (moderate or high suitability) for *S. intermedius* aquaculture from November to the next April (Fig. 4). Our previous study indicates that small *S. intermedius* (3 cm in test diameter) transplanted to Fujian in November grew to the market size (~ 5.5 cm in test diameter) in the next May^51^. Furthermore, the abalone and kelp aquaculture are well developed in Fujian province and the unused abalone cages and adequate feed supply can be used well for the aquaculture of *S. intermedius*^51,52^. Therefore, the transplantation of *S. intermedius* to the south is a promising method for the aquaculture expansion of *S. intermedius* in China, provided that the industrial high water temperature bottleneck can be addressed. According to the present study, a preliminary judgment can be made on whether a site is suitable for the expansion of *S. intermedius* aquaculture. More factors need to be taken into consideration before field application.

## Conclusion

Based on the SST data and the geographic information of the native species *A. japonicus* and the exotic species *S. intermedius*, we established an ecological niche model MaxEnt for the two aquacultural species. According to the present assessment, there is a great potential for the aquaculture expansion of *A. japonicus* in the north, especially the coastal areas of Hebei province and Tianjin. Furthermore, large expansion of *A. japonicus* aquaculture to the south is promising, since there are a number of suitable sites besides Xiapu. The present study indicates that ecological niche model can be used as a tool to assess the suitable sites for the aquaculture expansion of the native species in China. The present study highlights that high water temperature in July and August is the bottleneck for the expansion of *S. intermedius* aquaculture and this well explains the limited aquaculture expansion of *S. intermedius* in China. A new aquaculture model is suggested to break the high-water temperature bottleneck. Furthermore, the transplantation of *S. intermedius* to the south is a promising method for the aquaculture expansion. We propose that it is important to establish ecological niche models for the exotic species before they are introduced. The present study establishes a new tool for the preliminarily assessment of site suitability for the aquaculture expansion of sea cucumbers and sea urchins in China using the ecological niche model MaxEnt based on the SST data.

## Data Availability

GBIF data is available from https://www.gbif.org/. The data of sea surface temperature is available from https://neo.sci.gsfc.nasa.gov/.
